# Coaching tailored by stages: A valuable educational strategy to achieve independence in research

**DOI:** 10.1017/cts.2021.804

**Published:** 2021-06-18

**Authors:** Karen G. Martínez, Juan Carlos Jorge, Carlamarie Noboa-Ramos, Estela S. Estapé

**Affiliations:** 1 Department of Psychiatry, School of Medicine, Medical Sciences Campus, University of Puerto Rico, San Juan, 00936, Puerto Rico; 2 Department of Anatomy and Neurobiology, School of Medicine, Medical Sciences Campus, University of Puerto Rico, San Juan, 00936, Puerto Rico; 3 Department of Surgical Sciences, School of Dental Medicine & Tracking and Evaluation Core Leader, Hispanic Alliance for Clinical and Translational Research, Medical Sciences Campus, University of Puerto Rico, San Juan, 00936, Puerto Rico; 4 Senior Advisor, Post-doctoral Master of Science in Clinical and Translational Research, Medical Sciences Campus, University of Puerto Rico, San Juan, 00936, Puerto Rico and Director, Research Center, San Juan Bautista School of Medicine, Caguas, 00726, Puerto Rico

**Keywords:** Coaching, low-resource institutions, research environment, research development programs, research leadership

## Abstract

This study presents an individualized coaching approach tailored to the stages of proximity of promising scientists interested in becoming independently funded researchers in the context of a minority-serving institution. This strategy defined the participant’s stage of proximity by their number of first-author publications in peer-reviewed journals and their track record in submitting research grants. We argue that coaching tailored by stages is an asset to maintain the enthusiasm, persistence, and positive attitude of promising scientists as they try to reach independent investigator status. Furthermore, this valuable educational approach supports the development of management and leadership skills in defined scientific domains.

## Rationale for Coaching by Stages

The need to focus on scientists who will strengthen research environments applies to every academic organization, from high-resource institutions (HRIs) to low-resource institutions (LRIs) [1]. The search for promising research leaders on whom to invest becomes especially critical in LRIs, where in addition to limited fiscal resources dedicated to research infrastructure and release time for research, there is a scarcity of independently funded investigators who can serve as role models [2]. Many of the LRIs in the USA are also minority-serving institutions or are in geographical areas that have historically received lower funding from the National Institutes of Health (NIH) [3]. Enhancing diversity in the clinical translational research workforce has been a continued issue of serious concern for the scientific community [4]. One of the most consistent modifiable factors to improve researchers’ retention from underrepresented groups is increasing the number of mentors from these groups and closing the gap in obtaining competitive awards [5–7]. Unfortunately, the research career development programs available in LRIs are usually time-limited, mentored-based, and do not offer the long-term support needed to establish an independently funded research career. We identified an opportunity to integrate coaching to help advance postdoctoral scientists in mentored research career development with specific performance targets in time-limited activities [8]. Our educational strategy used coaching tailored by stages, focusing on goal attainment and leadership development.

## Unmet Need

The University of Puerto Rico-Medical Sciences Campus (UPR-MSC) is a public minority-serving institution. As an LRI, it has maintained an active NIH grantee portfolio with increased Hispanics, multiple disciplines, and women in the research workforce [9]. In fact, in 2002, the UPR-MSC and Morehouse School of Medicine were the first two NIH R25 Clinical Research Education and Career Development (CRECD) programs funded in the USA [10]. The UPR-MSC postdoctoral Master of Science in Clinical and Translational Research (MSc) is a 2-year mentored research career development program focused on training multiple disciplines in health disparities research.

In 2010, the UPR-MSC received a U54 NIH grant to create a network among three academic health centers in Puerto Rico to address clinical and translational research capacity building: the Puerto Rico Clinical and Translational Research Consortium (PRCTRC) [11]. Both programs focused on mentoring for early-career research development and in primary research outcomes such as scientific presentations, peer-reviewed, and public domain publications, recognitions/honors, and attainment of externally funded research projects. Career outcomes included academic positions with research release time, industry/research leadership positions, integration of research with clinical practice, and becoming a research mentor.

Since its inception, both the MSc program and PRCTRC goals were designed as complementary catalytic forces to enhance investigators’ competitiveness to conduct clinical and translational research through NIH R-type funding. Although these programs have successfully spearheaded early investigators’ research careers, both programs found that it was difficult to transition them into independent funding. Coaching by stages was considered a novel method to attend to this need, understand the barriers to independent research careers, and develop strategies to address them.

## Target Audience

The PRCTRC Professional Development Core (PDC), in collaboration with the Evaluation Core (EC), joined to design a strategy aimed to enhance competitiveness by increasing the number of publications and decreasing the time taken for the first NIH R-type grant application. The initiative was named *Independent Research Professional Development (IRPD) Fellowship.* The IRPD Fellowship was open to the 69 participants of the two PRCTRC main capacity-building activities: grantsmanship (*n* = 49), pilot projects (*n* = 17), and three that participated in both. The IRPD program recruited candidates with an interest in being coached to increase their research productivity. An initial email invitation was followed by two orientation sessions and the formal application submission within 2 months. A total of 35 candidates (51%) attended the first activity, of which 16 (23%) participated in the second one designed as an introduction to the IRPD program. Through a survey, participants noted that the IRPD program did not provide support for release time nor funds for research. These reasons may explain why only six candidates completed the IRPD Fellowship application.

The IRPD application consisted of a one-page letter of intent describing the candidate’s stage of proximity for independent funding, scientific goals, and commitment to participate in the program, a letter of support from a mentor/collaborator, and their biographical sketches. Applicants self-defined their stage of proximity for independent funding. The program had a maximum capacity of 10 IRPD fellows. The priority in the ranking for admission was early-stage investigators, those within 10 years of completing their terminal research degree or within 10 years of completing their medical residency, and then any graduate from a formal research degree or training program. Four of the six applicants were UPR-MSc graduates, of which one withdrew his application for personal reasons. Of the five final fellows, four were female, four were Puerto Rican, three were PhDs and two MDs.

## Description of the IRPD Fellowship

The IRPD Fellowship program consisted of at least 2 years of coaching depending on the Fellow’s Individual Development Plan, stage of proximity to become an independent researcher, their commitment to the program, and their annual productivity outcomes. The three coaches were local senior researchers who participated in the design of the IRPD program. They matched the three different fellow’s stages of development, expectations, and goals according to their expertise. Each coach had salary support of 5% time effort and had no additional coaching training, except for the leadership planning meetings. The coaches met monthly with the fellows to guide the discussion on how to obtain the individual and group goals. Table 1 shows the coaching strategy tailored by stage, specifying the desired research profile of fellows and their coaches and their expected outcomes.

**Table 1.** Coaching tailored by stage of proximity

Stage	Fellow	Individualized coaching*	Coach
**I**	Scientists with proven research productivity, defined as lead authorship in peer-reviewed publications and experience in grant submissions	Submission and attainment of R-type grants	An established independent investigator with recent NIH- RO1 funding and a strong publication record
**II**	Scientists with moderate lead authorship in peer-reviewed publications and experience in the submission of pilot projects	Submission of first, second, or corresponding author manuscripts in a defined NIH research area to enhance a competitive R-type grant application	A senior investigator with mentoring experience, portfolio of funded R-type grants, and strong publication record
**III**	Scientists who have been supporting authors without having led a publication as first or second author and no experience in grant submissions	Submission of first, second, or corresponding author manuscripts to enhance a competitive pilot project application	A senior investigator with mentorship experience in writing manuscripts and a strong publication record

* Attainment of research leadership skills by the fellow across Stages I–III.

The stage of the proximity of the fellows’ preparedness to submit an independent competitive research award was the critical factor for identifying the coach. Coaches did not need to have expertise in the fellow’s specific area of research and completed the match depending on the fellow’s goals and objectives. The stage’s characteristics determined the program’s priority in planning activities for each fellow and the design of expected outcomes. IRPD fellows identified the following core goals:

- To better understand review criteria and study sections.
- To participate in mock reviews.
- To better incorporate reviewers’ recommendations in the development or re-submission of a grant application.
- To develop strategies for nurturing collaborations with external experts.
- To better understand NIH Funding Opportunity Announcement (FOA) and Request for Proposals (RFPs) and their Institutes’ goals.
- To engage in peer-review of manuscripts and grant applications outside their research expertise.
- To sharpen manuscript writing according to peer-review guidelines.
- To implement an Individual Development Plan (IDP) as a tool to help, support, plan, and track career development and learning opportunities.
- To better understand the research infrastructure and procedures within the institution.
- To better comprehend academic advancement as a researcher.
- To improve negotiation skills with key institutional stakeholders to strengthen career as an academic researcher.

## Methods of Evaluation and Assessment

The first 2 years for each fellow consisted of individual coaching and participation in training, workshops, seminars, and workgroups to help them achieve their identified goals. After completing the first year, the stage of proximity and the specific targeted outcome for each fellow varied according to their level of research productivity. The EC examined the progress for the achievement level to further each individual professional planning by conducting a semiannual progress report and an annual online survey.

During the program, coaches guided the fellows to identify challenges and barriers limiting their research accomplishments. Fellows most frequently identified as barriers: limited resources (*n* = 3) such as funding support, editing support, technical equipment, human resources (study coordinator and administrative support), support from supervisors to release/protected time to conduct research (*n* = 3), a healthy work-life balance (*n* = 3), lack of preliminary data (*n* = 2), and lack of research experts (*n* = 1). Coaches guided fellows to identify solutions within the institution.

Table 2 presents the fellows’ outcomes 2.5 years after completion of the IRPD Fellowship program (from January 2017 to June 2020) according to their stage of proximity to become an independent investigator. Within this period, these five scientists co-authored 42 peer-reviewed publications. In addition, they submitted 16 external grant applications (75.0% NIH R-type grants), of which 7 received funding resulting in over 2.8 million dollars in external funding. Three fellows were promoted in academic ranking, one Associate Professor and two Full Professors. One fellow was promoted as a Chair of a Department and granted a tenure-track faculty position. A fellow was hired as Translational Clinical Trials Coordinator in an academic foundation specializing in cancer clinical trials. A fellow joined the San Juan Veterans Medical Center as a Principal Investigator of a multicenter Merit Review grant.

**Table 2.** Research outcomes after coaching tailored by stage

Stage	Criteria for proximity stage	Expected outcomes	Research outcomes
**I (*****n*** = **2)**	≥4 first, second, or corresponding author peer-reviewed publications within the last 5 years.	a) Submit ≥1 first, second, or corresponding author manuscript to peer-review journals.	11 publications as the first, second, or corresponding author 5 publications as co-author
	Has submitted ≥ 1 external competitive grant (not a pilot project).	b) Submit an independent R-type grant application as principal investigator.	14 grants submitted 6 grants awarded
**I (*****n*** = **2)**	≥3 publications in peer-reviewed journals within the last 5 years irrespective of author’s contribution.	a) Submit ≥ 2 first, second, or corresponding author manuscripts to peer-review journals.	9 publications as first, second or corresponding author 8 publications as co-author
	Has submitted ≥1 local or national pilot project.	b) Submit a competitive research grant application as principal investigator.	2 grants submitted 1 grant awarded
**III (*****n*** = **1)**	Does not meet the criteria established for Stage 1 or 2.	a) Submit ≥ 3 first, second, or corresponding author peer-reviewed publications.	1 publication as the first, second, or corresponding author 8 publications as co-author
		b) Submit a local or national pilot project as principal investigator.	1 clinical research site PI

Aside from these immediate outcomes, our coaching program has had long-lasting effects. For instance, these fellows continue as mentors to their graduate, medical and resident trainees, as directors in key institutional positions, as coordinators in interinstitutional academic research projects or the private sector. They also became peer-reviewers for leading journals in their respective fields and coaches of other postdoctoral investigators to secure future generations of clinical and translational researchers.

## Initial Evidence of Impact

Creating effective career development programs is instrumental for intensive research institutions to accelerate the research careers of researchers. Models for junior investigators’ career success include critical components, such as self-awareness, selecting the right topic, adequate support, working with others, and managing themselves [12]. Moreover, mentored research training during doctoral training leads to an increased likelihood of developing a research career [13]. These same models apply to Institutions of Emerging Excellence such as LRIs and minority institutions, but the need for a more assertive role model and coaching support is critical.

The IRPD Fellowship added the dimension of coaching postdoctoral scientists with previous mentoring experiences. After this experience, the coaches expressed the following three recommendations: 1) to safeguard protected research time for fellows (*n* = 3), 2) to increase institutional support to create a collective vision on how to support research career development efforts (*n* = 2), and 3) to ensure communication with the fellow’s mentoring and advising teams (*n* = 1).

**Fig. 1.**
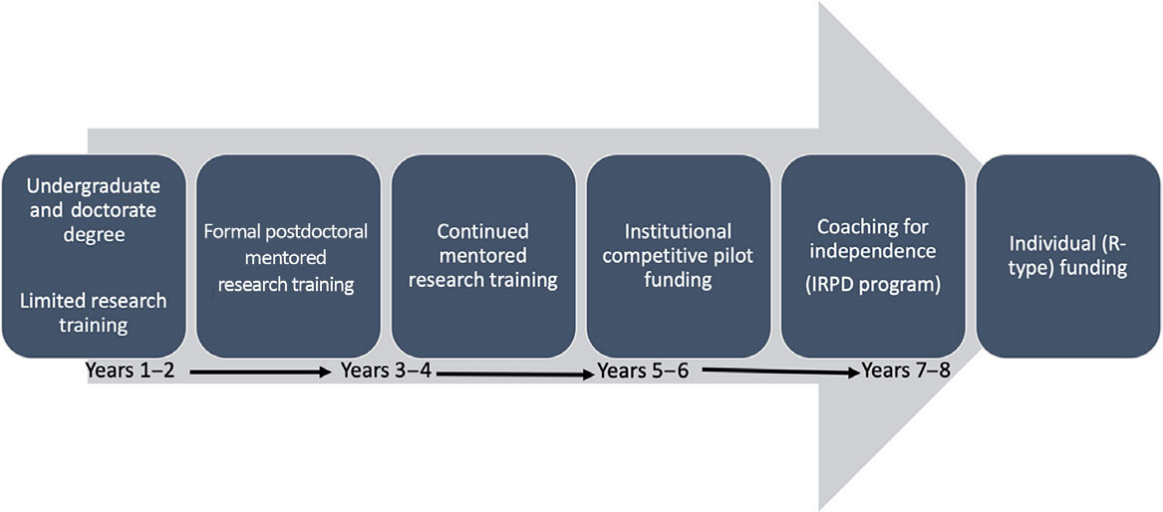
Postdoctoral research career development pipeline to achieve independence in clinical and translational research at low-resource institutions. The first years of training took advantage of advisors and mentors for role modeling and teaching on how to conduct clinical and translational research work. The Independent Research Professional Development (IRPD) Fellowship program incorporated coaching to identify and effectively address barriers toward independence.

The coaching strategy was novel in that it was implemented according to the fellow’s own stated goals and their stage of proximity to become an independent investigator. We understand that these approaches were critical to the success of this strategy. This model of incorporating coaching on career research development at the UPR-MSC can be a valuable tool for other low-resource research settings. Figure 1 presents a career pathway designed for minority postdoctoral researchers from mentored formal research training to up to 8 years, including coaching at the final stages to reach independent funding. The additional years of coaching are essential to identify and address the barriers toward independence identified by the fellows. Although we recruited only one cohort of IRPD fellows, this coaching model has been incorporated in the institution’s clinical and translational research career development program portfolio.

Many mentor development activities within clinical translational science programs have successfully incorporated coaching and leadership development [14,15]. Our experience shows that coaching was a valuable educational strategy for our researchers who had not reached independence to identify their strengths and limitations and develop a problem-solving approach to their research career advancement barriers. The mentored research experiences provided them with knowledge on grantsmanship in their research areas, but coaching offered guidance on applying that knowledge in their institutions. Awareness of institution-specific strengths and weaknesses is of utmost importance to break down institutional silos while strengthening and reinforcing institutional practices toward better research environments. We believe that a selected group of scientists serving as research leaders in LRIs can effectively create new institutional habits, behaviors, and practices to enhance a research culture.

Therefore, we strongly support that coaching by stages of proximity to become a funded investigator is an asset to maintain the enthusiasm, persistence, and positive attitude of promising scientists as they try to reach independent investigator status. This strategy also supports the development of management and leadership skills in their scientific domains.
